# Is temperature an important variable in recovery after mild traumatic brain injury?

**DOI:** 10.12688/f1000research.12025.1

**Published:** 2017-11-20

**Authors:** Coleen M. Atkins, Helen M. Bramlett, W. Dalton Dietrich

**Affiliations:** 1 Department of Neurological Surgery, The Miami Project to Cure Paralysis, University of Miami Miller School of Medicine, Lois Pope LIFE Center, 1095 NW 14th Terrace (R-48), Miami, FL, 33136-1060, USA

**Keywords:** concussion, fluid-percussion brain injury, hyperthermia, hypothermia, temperature, traumatic brain injury

## Abstract

With nearly 42 million mild traumatic brain injuries (mTBIs) occurring worldwide every year, understanding the factors that may adversely influence recovery after mTBI is important for developing guidelines in mTBI management. Extensive clinical evidence exists documenting the detrimental effects of elevated temperature levels on recovery after moderate to severe TBI. However, whether elevated temperature alters recovery after mTBI or concussion is an active area of investigation. Individuals engaged in exercise and competitive sports regularly experience body and brain temperature increases to hyperthermic levels and these temperature increases are prolonged in hot and humid ambient environments. Thus, there is a strong potential for hyperthermia to alter recovery after mTBI in a subset of individuals at risk for mTBI. Preclinical mTBI studies have found that elevating brain temperature to 39°C before mTBI significantly increases neuronal death within the cortex and hippocampus and also worsens cognitive deficits. This review summarizes the pathology and behavioral problems of mTBI that are exacerbated by hyperthermia and discusses whether hyperthermia is a variable that should be considered after concussion and mTBI. Finally, underlying pathophysiological mechanisms responsible for hyperthermia-induced altered responses to mTBI and potential gender considerations are discussed.

## Introduction

Mild traumatic brain injuries (mTBIs) and concussions occur in about 100 to 300 people per 100,000 annually worldwide ^1^. This public health problem is underestimated because an estimated 50% of people with a concussion do not seek medical attention ^2^. This places the annual incidence of mTBI and concussion at about 42 million people worldwide ^3^. The current definition of concussion as recommended by the 4th International Conference on Concussion in Sport in 2012 is “concussion is a brain injury and is defined as a complex pathophysiological process affecting the brain, induced by biomechanical forces” ^4^. This dysfunction is mediated by a cascade of pathophysiological responses to the injury that ultimately alters cerebral function without necessarily causing concurrent macrostructural damage ^4^. For the purposes of this review, we will use concussion and mTBI synonymously, as a non-penetrating head injury that alters brain functioning with no or brief loss of consciousness ^5^. Other than management of symptoms, the patient with mTBI is typically recommended to rest until symptoms resolve, followed by a gradual increase in return to normal activity ^6^. Concussions and mTBIs are a significant clinical problem yet lack effective evidence-based therapeutics ^2^.

Concussions and mTBIs can result in significant cognitive, psychosocial, and physical issues, although many of these symptoms resolve rapidly. Clinical assessments of learning and memory ability have revealed that a single concussion can result in significant impairments in working memory, attention and concentration issues, and decreased processing and reaction time ^7^. However, several studies have demonstrated that mTBI may also result in chronic cognitive impairments lasting for weeks after injury ^2, 8, 9^. Additionally, many have more chronic physical symptoms such as headaches, vestibular issues, irritability, depression, and fatigue, collectively referred to as post-concussion syndrome ^10– 12^. Although there is some variability in the recovery trajectory, for 50%–60% of individuals with mTBI, symptoms will typically resolve in 7 to 10 days ^8^. However, imaging and more objective clinical assessments indicate that abnormalities in the brain may persist beyond 30 days after mTBI ^13^.

## Prevalence of hyperthermia during mild traumatic brain injury

An aspect of mTBI that has been understudied and may affect recovery is the effect of temperature. It is well known in severe and moderate TBI that brain temperature has a profound effect on outcome ^14, 15^. If the patient is hypothermic, outcome is improved, but if the patient is hyperthermic, outcome is greatly worsened. However, hyperthermia has not been typically considered to be an important variable in mTBI studies despite the potential co-occurrence of hyperthermia with mTBI.

**Figure 1.**
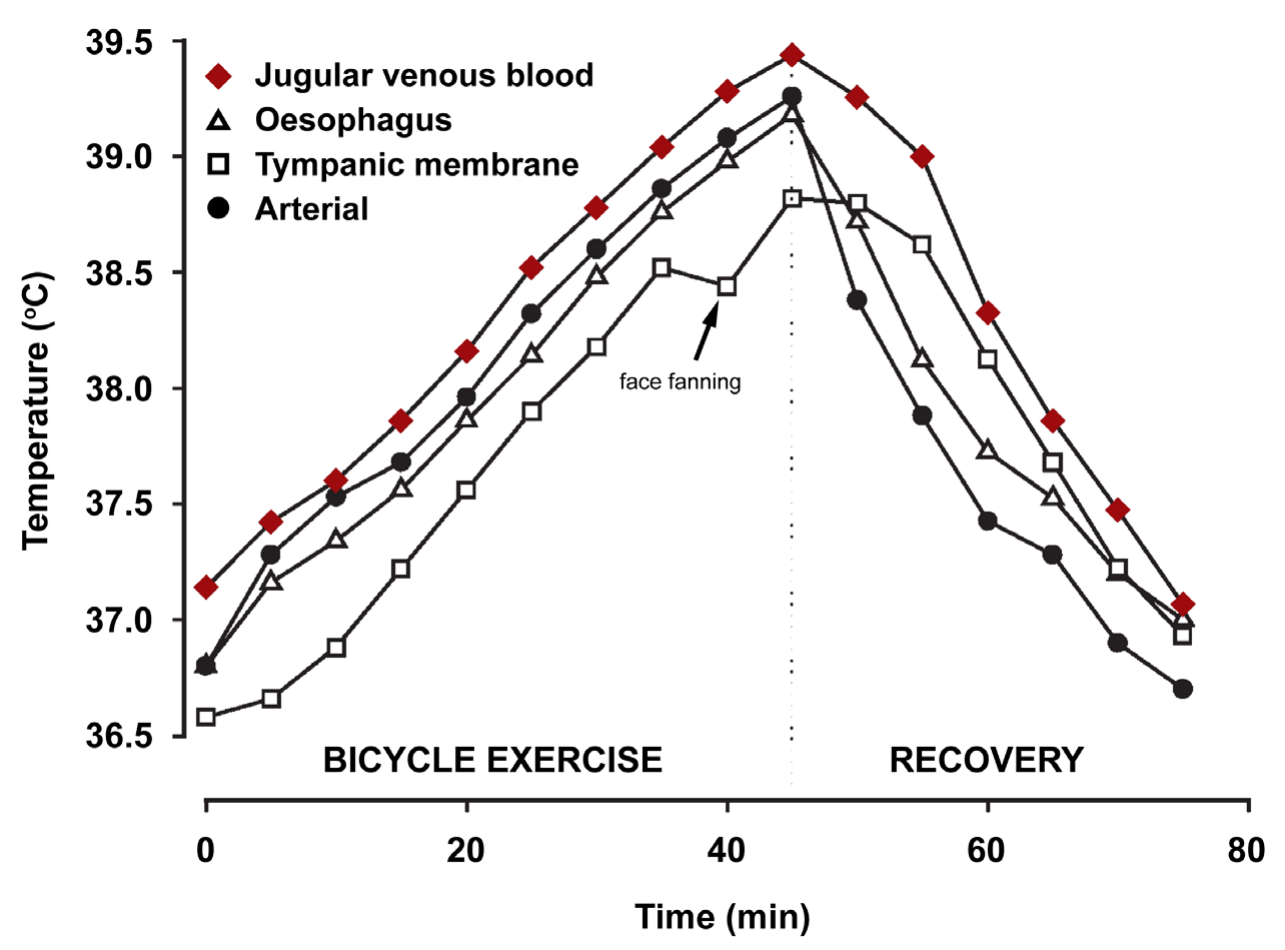
Temperature responses during cycling. Jugular venous blood temperature most closely reflects brain tissue temperature and is still elevated at 60 minutes during recovery (arrow). Figure reproduced with permission ^20^.

Many athletes experience mTBIs in the context of mild hyperthermia. Strenuous exercise can raise core temperature to 39–40°C, and this temperature increase is more long-lasting in hot environments ^16– 18^. Exercise-induced hyperthermia slowly recovers once an athlete has stopped exercising ^19, 20^. If core temperature has risen to 39–40°C, this temperature naturally returns to normothermic levels within 60 minutes to 3 hours after exercise has ceased ^19, 21^. The brain in particular is slow to cool, and published studies have found that after 1 hour of bicycle exercise, the jugular venous blood, a close approximation of brain temperature, is still significantly hyperthermic even 60 minutes after exercise has ceased ( Figure 1) ^20^.

Another contribution to hyperthermia during mTBI is a hot ambient environment. Military personnel in Iraq and Afghanistan, countries with high environmental temperatures, are at risk to be hyperthermic when injured ^22^. The combination of high exertion and warm humid environments has the potential to raise the risk of experiencing mTBI while hyperthermic even further ^16– 18^. Thus, an mTBI experienced in the context of hyperthermia can be a common occurrence due to high environmental temperatures and, in particular, among people who are engaged in activities with a high relative risk for head injury.

Only a small proportion of energy produced by muscle metabolism is used by work, whereas over 75%–80% of the energy released by muscle metabolism is heat ^23^. Evaporation is effective in dissipating large amounts of heat and this limits core-temperature increases to 2–3°C in trained athletes ^18, 24^. Despite these natural cooling mechanisms, increases in core temperature to 39–40°C are commonly observed in people because of high-intensity activity ^18, 25^. Heat dissipation is also slowed by the environment temperature. Adaptation is well known to occur with exercise in warm environments and these physiological adaptations improve performance, but core temperature still rises to mildly hyperthermic levels with exercise in heat-adapted individuals ^23, 24^.

The brain is not protected against increases in core temperature and, in fact, stays warmer for a longer period of time after a rise in core temperature to hyperthermic levels ^21^. Exercise increases cerebral temperature because physical activity results in an increase in heat production of the muscles, which elevates arterial blood temperature. Heat removal from the brain is mediated by the heat capacity of the blood, cerebral blood blow, and the arterio-venous blood temperature difference. Increased arterial blood temperature elevates cerebral temperature at the same rate as the rise in core temperature ^20^. However, owing to the limited heat removal capacity of the brain, brain temperature returns more slowly than core temperature and is still hyperthermic for up to 60 minutes with passive rest after exercise ^20^.

Assessing brain temperature after an mTBI is complicated by the dissociation of brain temperature from oral, rectal, or tympanic membrane temperature ^26, 27^. Indeed, these temperature readings can be 1–3°C lower than brain temperature ( Figure 1). Temperature measurements from alternative locations such as the temporal artery more accurately reflect brain temperature but are not commonly used ^26, 27^. Thus, brain hyperthermia may be a likely variable at the time of mTBI, but studies assessing this variable in patients with mTBI are lacking.

## Effects of hyperthermia

**Figure 2.**
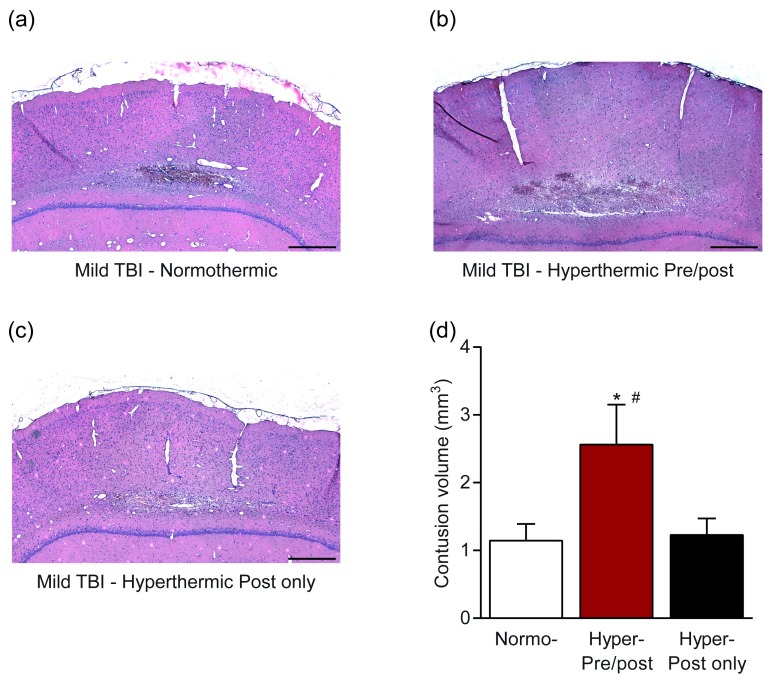
Exacerbation of cortical contusion volume with hyperthermia after mild traumatic brain injury (TBI) in an experimental model of TBI in rats. Animals received mild fluid-percussion brain injury while ( **a**) normothermic (37°C), ( **b**) hyperthermic (39°C) beginning 15 minutes prior to mild TBI and for 2 hours after injury, or ( **c**) hyperthermic (39°C) only for 2 hours after injury. ( **d**) Brains were sectioned and stained with hematoxylin and eosin to visualize and quantify cortical contusion volume. Scale bars = 300 µm. * *P* <0.05 versus normothermic, ^#^ *P* <0.05 versus hyperthermic post only. Adapted from 32.

**Figure 3.**
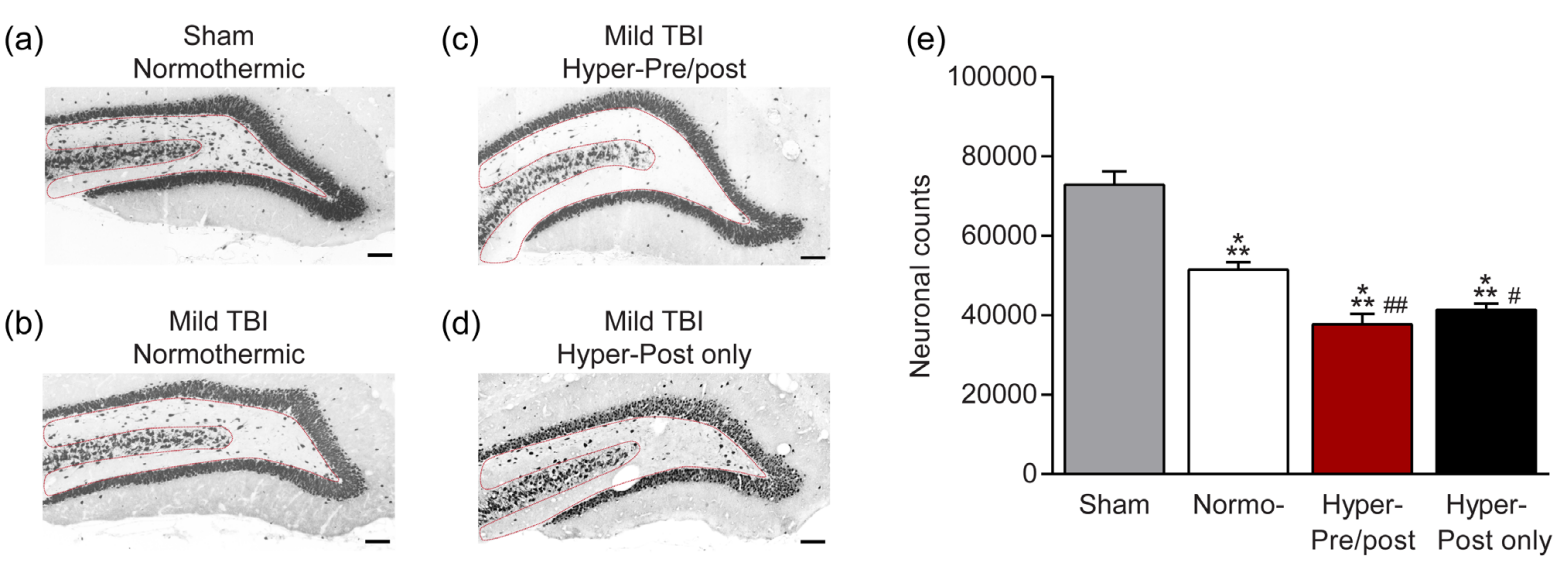
Increased hippocampal cell loss after mild traumatic brain injury (TBI) and mild hyperthermia. Animals received ( **a**) sham surgery, mild fluid-percussion brain injury while ( **b**) normothermic (37°C), ( **c**) hyperthermic (39°C) beginning 15 minutes prior to mild TBI and for 2 hours after injury, or ( **d**) hyperthermic (39°C) only for 2 hours after injury. ( **e**) Neuronal loss in the dentate hilus was quantified by stereology in NeuN-immunostained sections. Scale bars = 300 µm. *** *P* <0.001 versus Sham, ^#^ *P* <0.05, ^##^ *P* <0.001 versus normothermic. Adapted from 32.

The detrimental consequences of post-traumatic hyperthermia following more moderate and severe TBI have been reported by multiple laboratories and previously summarized ^14, 28^. In various animal models of TBI, induced periods of mild hyperthermia at various post-traumatic time periods significantly aggravate histopathological and behavioral outcomes ^29^. In clinical investigations where severely brain-injured patients have been monitored, periods of fever are common and also result in worsening of functional outcomes ^30^. In contrast to these findings, the consequences of hyperthermia on mTBI and concussion symptoms and recovery are a fairly new and active area of investigation ^31^. To address this important question, we determined whether hyperthermia would also affect pathological and cognitive outcome after mTBI. We used a model well established to recapitulate several aspects of human mTBI, mild fluid-percussion brain injury, where saline is briefly pulsed on the dura of the parietal cortex. Using this preclinical model of mTBI in rats, mild fluid-percussion brain injury, we found that mTBI in the context of mild hyperthermia (39°C) both pre- and post-injury resulted in a significant exacerbation of pathology ( Figure 2) as assessed by cortical contusion volume ^32^. The mTBI pathology converted into pathology that was nearly equivalent to the pathology observed in moderate TBI. However, hyperthermia after, but not prior to or during, mTBI did not worsen cortical contusion volume. The exacerbation of pathology due to hyperthermia also occurred in the hippocampus, and increased neuronal loss occurred in mTBI animals that were hyperthermic both pre- and post-injury ( Figure 3). Furthermore, hyperthermia in only the post-injury period also increased hilar neuronal loss. Thus, preclinical studies demonstrate that mTBI experienced while hyperthermic significantly exacerbates neuronal loss and cortical damage.

**Figure 4.**
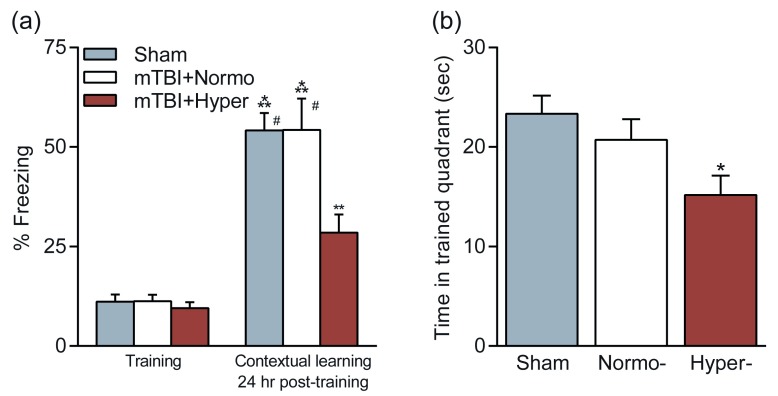
Effects of temperature manipulations on cognitive outcome after mild traumatic brain injury (mTBI). Animals received mild fluid-percussion brain injury while normothermic (37°C, mTBI+Normo) or hyperthermic (39°C, mTBI+Hyper). Cognition was assessed at 2–3 weeks post-injury and compared with non-injured, sham animals. ( **a**) Contextual fear conditioning was unaffected by mTBI but impaired in hyperthermic mTBI animals. ( **b**) Water maze performance on the probe trial to assess retention of spatial learning. Time spent in the trained quadrant was significantly decreased in hyperthermic mTBI animals as compared with sham animals. Figure reproduced with permission ^33^.

This conversion of pathology also resulted in an exacerbation of cognitive impairments ^33^. Animals that received mTBI when normothermic had no significant learning or retention deficits in contextual fear conditioning or the water maze ( Figure 4). However, animals that received mTBI while hyperthermic (39°C) had significant deficits in both hippocampal-dependent learning tasks as compared with animals that received mTBI during normothermic conditions or sham controls. Thus, a relatively mild elevation in brain temperature at the time of an mTBI causes hippocampal-dependent behavioral deficits. We tested whether reducing brain and body temperature to normal physiological temperatures 15 minutes after an mTBI would prevent the development of behavioral deficits. We found that animals that received an mTBI with mild hyperthermia but then were cooled to normothermic levels at 15 minutes post-TBI did not exhibit deficits in the water maze or with contextual fear conditioning and performed at sham, non-injured levels ( Figure 4). These results may have important implications in the treatment of mTBI for athletes in warm ambient environments. The simple treatment of cooling rapidly after mTBI has the potential to prevent the development of hippocampal-dependent learning deficits.

Although the current discussion focused primarily of elevated brain temperature after brain injury, it is known that elevations in brain temperature under stressful conditions may have important biological effects independent of injury mechanisms ^34, 35^.

## Temperature-sensitive biomarkers

The failure of multiple phase 3 clinical trials for TBI has directed National Institutes of Health and US Department of Defense working groups to conclude that non-invasive measures of therapeutic efficacy need to be developed to demonstrate that the therapy is engaging its proposed molecular target(s) and resulting in a biological effect ^36^. Determining whether a biomarker panel detects mTBI and is sensitive to temperature manipulations would greatly improve clinical guidance ^37, 38^. Efforts to develop biomarkers released from the injured brain and detected in the serum for mTBI have been hampered by the lack of sensitivity to concussion and the short half-life of most brain-derived biomarkers of mTBI ^39, 40^. Biomarkers sensitive to injury severity for TBI include glial fibrillary acidic protein (GFAP), which is released from injured astrocytes, and ubiquitin C-terminal hydrolase L1 (UCH-L1) ^41, 42^. However, both are limited in utility because of the relatively short half-life of the biomarkers. Other promising biomarkers that are being evaluated for their diagnostic utility for mTBI include auto-antibody to GFAP, auto-antibody to S100β, tau, select microRNAs (miRNAs), and plasma soluble cellular prion protein ^43– 47^. Preclinical and clinical studies have demonstrated that several of these biomarkers are sensitive to therapeutic hypothermia in moderate to severe TBI. In particular, GFAP and UCH-L1 are downregulated after therapeutic hypothermia in preclinical models of moderate TBI ^48, 49^. Therapeutic hypothermia decreases levels of GFAP in adult patients with severe TBI but not in pediatric patients with TBI ^50, 51^. However, both GFAP and UCH-L1 are limited in utility for mTBI because of the relatively short half-life of these biomarkers. Other potential biomarkers that are temperature-sensitive in moderate to severe TBI include neuron-specific enolase, S-100, brain-specific creatine kinase, and several miRNAs ^52– 54^. Whether they are regulated by temperature after concussion or mTBI remains to be established. Pro-inflammatory cytokines are highly sensitive to temperature manipulations after TBI, including hyperthermia ^55– 59^. However, cytokines are limited in utility for mTBI since mTBI often can be complicated by systemic injuries and these biomarkers do not differentiate well between brain-derived versus systemically derived sources. Biomarkers known to be sensitive to temperature in other studies include MMP-9 and HSP70 and merit study for mTBI ^60– 63^.

## Interaction of gender and temperature as critical variables for traumatic brain injury outcome

The importance of gender on traumatic and ischemic outcome has been documented in various models of brain injury ^64– 66^. In one study, female rats after moderate fluid-percussion injury (FPI) demonstrated significantly smaller contusion volumes compared with male rats ^66^. In that study following ovariectomy, contusion volumes in females were not significantly different than those in males. These and other data emphasize the importance of estrogen and progesterone in the pathogenesis of TBI ^64^. Recent studies have also demonstrated that alterations in post-traumatic temperature have gender-specific effects on outcomes after moderate TBI. For example, Suzuki and colleagues ^67^ reported that while post-traumatic hypothermia following moderate FPI provided neuroprotection, no significant effect on contusion volume was seen in female rats. Thus, there appear to be gender-specific effects of temperature manipulations in preclinical models of brain injury.

In reference to the present discussion, more recent studies have evaluated the effects of post-traumatic hyperthermia in models of moderate TBI. In this regard, Suzuki and colleagues ^66^ reported that an induced period of mild hyperthermia after moderate TBI increased contusion volumes, cortical neuronal cell death, and axonal damage in intact female rats. Interestingly, in that study, the effects of post-traumatic hyperthermia were more pronounced in ovariectomized animals. These results therefore emphasize that hyperthermia worsens outcomes after moderate TBI in female rats and that neural hormones may protect against secondary hyperthermic insults. Future studies are required to determine whether gender is also an important factor in the pathogenesis of mTBI or concussion and the detrimental effects of hyperthermia on functional outcomes.

## Underlying temperature-sensitive pathomechanisms and future research directions

The pathophysiology of TBI is complex and involves multiple injury cascades that have been reported to be temperature-sensitive ^14, 28, 68^. In regard to post-traumatic hypothermia, numerous reports have shown that lowering brain temperature after a moderate to severe TBI reduces excitotoxicity, free radical generation, apoptosis, and neuroinflammation ^28, 69– 71^. In the area of inflammation, post-traumatic hypothermia after moderate FPI reduces blood-brain barrier permeability to both large and small tracers and the infiltration of CD68-positive cells ^72^. Other studies have also shown that whereas hypothermia reduced the accumulation of polymorphonuclear leukocyte infiltration after TBI, post-traumatic hyperthermia increased inflammatory cell accumulation ^73^. These studies are also in agreement with post-traumatic temperature modifications (for example, that periods of hyperthermia significantly alter levels of several pro-inflammatory cytokines and inflammasome proteins) ^74^.

Most recently, the effects of post-traumatic hypothermia on microglial and macrophage phenotype polarization have been investigated after moderate TBI ^75^. In that study, temperature-sensitive effects on the various subsets of pro-inflammatory (M1) and anti-inflammatory (M2) microglia and macrophages were determined by using flow cytometry and reverse transcription–polymerase chain reaction. This study provided a link between temperature-sensitive alterations in macrophage/microglia activation and polarization toward an M2 phenotype with hypothermia that could be permissive for cell survival and repair. Studies to determine whether mild hyperthermic TBI also alters microglial and macrophage polarization are under way. Taken together, these studies emphasize the importance of post-traumatic temperature on pro-inflammatory signaling after brain injury.

Diffuse axonal injury (DAI) is a common consequence of mild and moderate TBI and is considered to be significantly related to the resulting functional deficit ^76– 78^. Previous studies have reported the importance of post-traumatic temperature in the frequency of damaged axons by using a variety of immunocytochemical techniques such as beta-amyloid precursor protein (β-APP). For example, after moderate TBI, hypothermia reduced the frequency of axonal damage at early post-traumatic time periods in several animal models ^79^. In reference to the effects of hyperthermia on DAI, Suzuki and colleagues ^66^ reported that post-traumatic hyperthermia after moderate TBI significantly increased axonal damage as indicated by β-APP in both intact and ovariectomized female rats. Following mTBI or concussion, evidence for white matter perturbations has been reported in animal models as well as clinical investigations ^80– 82^. Future studies are required to determine the effects of hyperthermia on DAI in models of mTBI and concussion.

An important cause of persistent behavioral problems after mTBI is pituitary dysfunction. Hypopituitarism has a prevalence rate of 25%–50% of patients with TBI overall and was found to occur in 37.5% of patients with mTBI ^83, 84^. Whether temperature at the time of injury alters this prevalence rate has yet to be studied. Interestingly, in an animal model of mTBI, core-temperature regulation was found to be disrupted in the post-injury recovery period during exercise ^85^. Understanding the interaction of pituitary dysfunction and temperature at the time of mTBI may facilitate clinical guidance in identifying which patients with mTBI need to be screened for potential pituitary dysfunction.

## Conclusions

mTBI or concussion is a serious and fairly common medical problem that can affect all age groups and produce both short- and long-term consequences. Because of the high incidence of concussion in athletes and military personnel, a great appreciation of the detrimental effects of single and multiple concussions has led to more research on this particular type of brain injury. Recent studies have implicated gray and white matter pathologies that result in a spectrum of neurological symptoms. Complicated concussions that lead to long-term disturbances in memory function or other problems require patients to undergo extensive rehabilitation strategies to retrain the nervous system. In parallel, new treatment strategies that target selective neurotransmitter systems and secondary injury mechanisms are being tested in animal models and in some clinical studies.

It now appears from experimental and clinical work that small alterations in brain temperature at the time of an mTBI or concussion may be among several factors that can affect the brain’s response to injury through altering secondary injury mechanisms. Therefore, mild levels of hyperthermia that can occur in people during periods of strenuous activity or exercise may be considered a risk factor for more severe or long-term functional problems. In regard to potential treatment strategies, normalizing brain temperature after a hyperthermic brain injury appears to reduce the degree of structural damage and reduce behavioral outcomes. In this regard, new technologies are being used to develop cooling helmets to reduce brain temperature in acute injury settings such as brain trauma. Also, established targeted temperature management approaches currently being used in severely injured patients should be considered for individuals with milder brain injuries. In the future, new pharmacological strategies that target specific injury cascades that have been shown to be aggravated with elevated temperature need to be identified and tested in clinically relevant concussion models. In this regard, it might be reasonable to consider first testing US Food and Drug Administration–approved drugs that have shown some promise in clinical studies and trials for severe TBI. Only through continued research will new prevention and treatment strategies be discovered and tested to minimize the potentially devastating consequences of mTBI and concussion.
